# Garden-Based Integrated Intervention for Improving Children’s Eating Behavior for Vegetables

**DOI:** 10.3390/ijerph17041257

**Published:** 2020-02-15

**Authors:** Seon-Ok Kim, Sin-Ae Park

**Affiliations:** 1 Department of Bio and Healing Convergence, Graduate School, Konkuk University, Seoul 05029, Korea; kso0804@naver.com; 2 Department of Environmental Health Science, Sanghuh College of Life Science, Konkuk University, Seoul 05029, Korea

**Keywords:** eating habits, gardening, horticultural therapy, urban agriculture, mediating factors

## Abstract

This study was conducted to develop and verify the effects of a garden-based integrated intervention for improving children’s eating behavior for vegetables. A pre-pos*t*-test experimental design was employed. The participants were 202 elementary school students (average age: 11.6 ± 1.5 years). The garden-based integrated intervention program was conducted during regular school hours for a total of 12 weeks. The program, based on a mediator model for improving children’s eating behavior, included gardening, nutritional education, and cooking activities utilizing harvests. In order to examine effects of the program, the mediating factors related to children’s eating behavior were evaluated using pre-post questionnaires. As a result of the program, dietary self-efficacy, outcome expectancies, gardening knowledge, nutrition knowledge, vegetable preference, and vegetable consumption were significantly increased, and food neophobia was significantly decreased. In addition, there were positive correlations between most mediating factors. Thus, the garden-based integrated intervention developed in this study was effective in improving children’s eating behavior for vegetables.

## 1. Introduction

Nutrition in growing children is very important; dietary habits that affect the nutritional state established in infancy and childhood, are solidified in adolescence [1]. The dietary life of elementary school students not only directly affects their health status at the time but is also linked to health status in adulthood and significantly affects lifetime health [2,3,4]. Incorrect eating behavior in childhood can lead to nutritional imbalances, growth disorders, obesity, metabolic syndrome, and various diseases in adulthood [5,6].

Nutritional status at school age affects not only physical aspects but also emotional and intellectual aspects [7]. According to Hinton [8], eating behavior in adolescence is related to psychological stability and character formation. Elementary school students are at the stage of the maturation of the cognitive ability to judge and synthesize objects, as well as self-concept formation; thus, development of the mind and body through a healthy diet is all the more important [9].

According to the Korea National Health and Nutrition Examination Survey, the average daily vegetable consumption of children aged 6 to 11 years is about 148 g, which is about one-third (420 g) the recommended daily vegetable consumption for this age group. As a result, more than half of all children in South Korea consumed less than the recommended amount of nutrients such as calcium and vitamin C, which are derived mainly from vegetables [10].

Accordingly, various methods such as school garden programs, nutrition education, or cooking activities have been attempted to improve children’s eating behavior worldwide [11,12,13]. In particular, gardening, in which children themselves plant, grow, and harvest vegetables, have been reported to reduce their revulsion toward vegetables and increase their preference for and understanding of various types of foods and leading to increased vegetable consumption [14,15,16,17,18,19]. In a previous study, as 304 students predominately Hispanic/Latino third- through fifth-grade in elementary school were randomly assigned to either the LA Sprouts group (nutrition, cooking, and gardening intervention) or control group, a number of determinants of dietary behaviors such as identification of vegetables and nutrition and gardening knowledge for LA Sprouts group has positively changed after the program [20]. Wells et al. [21] reported that the gardening program with nutrition and environment education improved fruit and vegetable availability of 2768 students in grades second through fifth. In the United States, the ‘Farm to School’ has shown positive results such as increased participation in school meals, increased choice, preference, and intake of fruits and vegetables, while conducting various activities for promoting, procuring, providing, and educating local foods in schools [22].

However, there are not enough studies to investigate the effect of garden-based integrated intervention and to analyze the mediating effects of factors affecting children’s eating behavior. Therefore, this study was conducted to develop and determine the effects of a garden-based integrated intervention program combining nutrition education and cooking activities, based on mediating factors, for improving the eating behavior to vegetables of elementary school students.

## 2. Materials and Methods

### 2.1. Participants

For recruitment of participants, a letter regarding the garden-based integrated intervention program was sent to elementary schools in the Seoul area, South Korea. Based on school garden environment, available grade levels, and number of students who agreed to participate, elementary schools C and K were selected. Promotional materials about the study and the program were sent to all parents of 3rd in the school C and 6th graders in the school K. Finally, the parents of all students provided consent for them to participate in the study (95 3rd graders in the school C and 107 6th graders in the school K). The average age of the participants was 11.6 ± 1.5 years. With regard to gender distribution by grade, there were 45 (47.4%) boys and 50 (52.6%) girls in the 3rd grade and 54 (50.5%) boys and 53 (49.5%) girls in the 6th grade. Before the program, the researcher visited the schools and conducted an orientation session in which the purpose of this study and schedule were described, precautions were listed, and written informed consent was obtained from participants. This study was approved by the Institutional Review Board of Konkuk University (7001355-201805-HR-242).

### 2.2. Garden-Based Integrated Intervention

#### 2.2.1. Program Development

**Figure 1:**
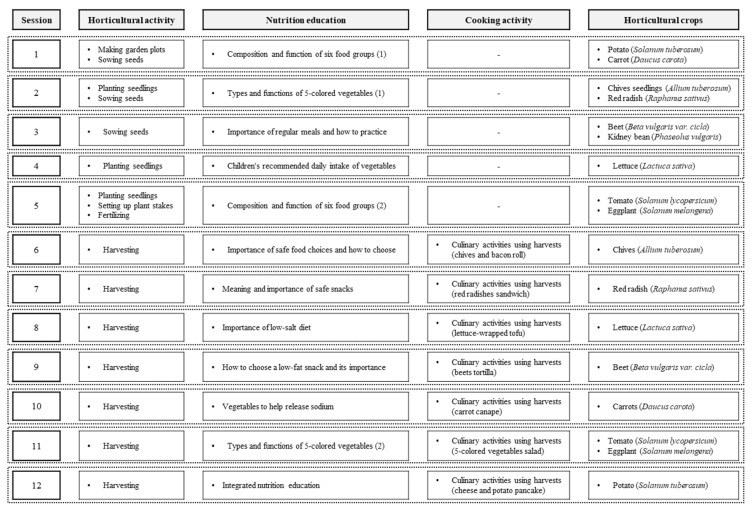
Development of the garden-based integrated intervention for improving children’s eating behavior.

A 12-session integrated intervention program, incorporating gardening, nutrition education, and cooking activities using harvests, was developed (Figure 1). This program was based on a mediator model for improving children’s eating behavior for vegetables, and these mediating factors were extracted from previous studies and sub-elements of social cognitive theory.

In order to select mediating factors affecting children’s eating behavior for vegetables, related studies were identified by using the following keywords in databases such as Web of Science, ProQuest Dissertation and Theses, Academic Search Premier, and Korean National Assembly Digital Library: (1) gardening-related keywords (horticultural activity, gardening, gardening program, garden therapy, allotment garden, urban agriculture, horticultural therapy, and therapeutic horticulture), (2) eating behavior-related keywords (vegetable, fruit, eating habit, eating behavior, dietary behavior, and nutrition), and (3) children-related keywords (children, kid, youth, and elementary school students). As per the search results, 40 research papers were used as reference to verify the effectiveness of the garden-based program to improve children’s eating behavior. The five factors that mediated the association between garden-based activity and children’s eating behavior for vegetables and fruits extracted from 40 studies were “skills required to engage in gardening” [23,24,25], “knowledge of nutrition, fruit, and vegetable” [19,26,27,28], “willingness to try new food (food neophobia)” [25,26,29], “dietary self-efficacy” [19,27,28], and “fruit and vegetable preference” [19,25,26,27,28,29].

**Figure 2:**
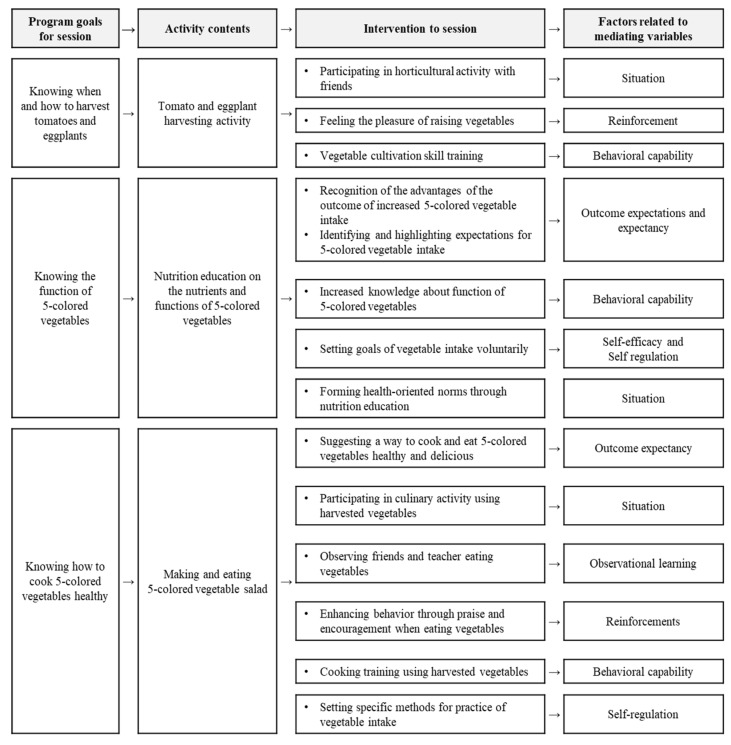
An example of the 11th session in the garden-based integrated intervention. Situation is individual’s perception of the physical and social environment and its environment; Reinforcement is praise, encouragement, and inner reward (pleasure of activity itself); Behavioral capability is knowledge and skills of the behavior; Outcome expectations are a person’s estimate that a given behavior will lead to certain outcomes; Outcome expectancy is belief about the consequences of one’s action; Self-efficacy is individual’s beliefs in their capabilities to perform a specific action required to attain the desired outcome; Self-regulation is ability to regulate and control their behavior; Observational learning is observing and following other’s behaviors [37].

Moreover, sub-elements of the social cognitive theory were incorporated in each session of the program according to the relevant themes (Figure 2). Social cognitive theory is widely used because it presents various ways of modifying behavior when planning and implementing health and nutrition education, as well as identifying factors related to eating behavior, and is especially used for nutrition education in children and adolescents [30,31,32,33]. The following elements of the social cognitive theory were applied in this program: outcome expectations, outcome expectancies, self-efficacy, situation, observational learning, reinforcements, behavioral capability, and self-regulation. Therefore, a total of 12 mediating factors were extracted from previous studies and sub-elements of the social cognitive theory were selected and incorporated in the garden-based integrated intervention.

The activities in the 12-session program were focused on gardening, nutrition education, and cooking activities using the harvests. The gardening activities mainly included plant cultivation, such as making plant beds, transplanting, watering, weeding, and harvesting plants. The participants cultivated seasonal plants, such as potato (*Solanum tuberosum*), lettuce (*Lactuca sativa*), tomato (*Lycopersicon esculentum Mill.*), eggplant (*Solanum melongena*), carrot (*Daucus carota*), and beet (*Beta vulgaris var. cicla*). Moreover, the gardening knowledge education was focused on the cultivation, harvesting, and management methods of each crop, referring to Unit 2-1 (Growing Plants in Everyday Life) of a 6th-grade Korean elementary school textbook [34].

Regarding nutrition education, the contents were selected by the Ministry of Food and Drug Safety and the Ministry of Education of Korea [35,36]. It was designed in connection with the contents of each horticultural activity to understand the relationship between diet and health and to acquire the ability to choose healthy foods. Cooking activities were carried out using harvested crops grown in the garden. By cooking and tasting the vegetables with their friends and teachers, students could learn how to cook vegetables healthier and improve observational learning and behavioral capability (Figure 1). Three researchers in horticultural education and horticultural therapy fields developed the intervention and then two elementary school teachers peer-reviewed.

#### 2.2.2. Program Operation

The 12-session garden-based integrated intervention was conducted once a week and 40 min per session. It was run by researchers who are a horticultural therapist certified by the Korean Horticultural Therapy Association and two volunteers during regular class hours. Gardening activities were conducted in the schools’ outdoor gardens, with a size of about 6 m^2^ for each class, while nutrition education and cooking activities were conducted in indoor activity rooms. After each session, parents were sent newsletters that included recipes using horticultural crops and nutrition education. Children attempted the recipes with their parents at home and shared photos of their activities through SNS (Social Network Services) with their friends and teachers. An example of the 11th session in the garden-based integrated intervention is presented in Figure 2.

### 2.3. Assessments

#### 2.3.1. Mediating Factors Related to Eating Behavior

In order to identify the mediating factors influencing children’s eating behavior, self-reported questionnaires were used before and after the program. A pilot test was conducted by 10 elementary students to determine the level of difficulty and suitable questions for elementary school students before starting the study.

Self-efficacy was assessed with a dietary self-efficacy questionnaire on fruit and vegetable consumption for elementary students [38]. A 24-item scale, with sub-domains such as whether to buy fruit and vegetable when going shopping with family and whether to choose fruit and vegetable at mealtime or snack time, was developed. The items were scored on a three-point Likert scale, with higher scores indicating higher dietary self-efficacy for fruit and vegetable consumption; the total score of the questionnaire ranged from 0 to 48. In this study, Cronbach’s alpha was 0.89 and 0.90 in the pre-program and post-program test, respectively.

To assess outcome expectancies for vegetable consumption, an outcome expectation questionnaire for children’s fruit and vegetable consumption, developed by Domel et al. [39], was modified for this study. The resulting 17-item scale included three sub-domains: social health, physical ability, and behavior. The items in this tool are scored on a three-point Likert scale, with negative outcome expectancies scored in reverse. Higher scores are indicative of higher outcome expectancies for children’s vegetable consumption and the total score of the questionnaire ranged from 0 to 34. In this study, Cronbach’s alpha was 0.78 and 0.82 in the pre-program and post-program test, respectively.

In order to assess aversion to new foods, the 10-item food neophobia scale developed by Pliner and Hobden [40] and translated into Korean by Choi [41] was used. While the original is scored on a seven-point Likert scale, in consideration of the present participants’ age, in this study, the questionnaire was scored on a three-point Likert scale. Items 1, 4, 6, 9, and 10 were reverse-scored, and the higher the total score, the higher the food neophobia; the total score of the questionnaire ranged from 0 to 20. In this study, Cronbach’s alpha was 0.78 and 0.68 in the pre-program and post-program test, respectively.

To investigate vegetable preferences, a list of representative vegetables from the Dietary Reference Intakes for Koreans 2015 was used [42]. Some of the 30 vegetables were extracted and beet, chive seedlings, and kidney beans were added; finally, 29 lists were used. Preference for each item was to be indicated on a six-point scale: “I don’t know,” “I really don’t like it,” “I don’t like it,” “So-so,” “I like it,” and “I really like it.” Higher scores were indicative of higher vegetable preferences and the total score of the questionnaire ranged from 0 to 145.

To assess nutrition knowledge, the textbook for Korean elementary students published by the Ministry of Food and Drug Safety and the Ministry of Education was referred to in order to select items that matched the educational contents of this program. The items were revised and supplemented after consultation with elementary school teachers. In order to classify the degree of difficulty by grade, 15- and 21-item were graded by referring to textbooks for 3rd graders and 6th graders, respectively (e.g., vegetables have a variety of roles depending on the color, among which green vegetables strengthen bones; the recommended amount of vegetables consumed per day for children in South Korea is 2 to 3 plates). Each nutritional knowledge item had three response options: “Yes,” “No,” or “I don’t know.” Participants received 1 point for a correct answer and 0 points for an incorrect answer. The perfect score of the 3rd students’ questionnaire is 15 points, and that of the 6th students’ questionnaire is 21 points. Higher scores were indicative of greater nutrition knowledge.

For the assessment of gardening knowledge, the 6th grade textbook of Korean elementary was referred to in order to select items that matched the educational contents of this program, and after consulting with an expert such as elementary school teachers and specialists in horticulture, the researcher revised and supplemented the scale. The final 10-item scale included questions about environmental factors necessary for plants to grow, knowledge about plant cultivation, and so on (e.g., What is non-root vegetable among carrot, potato, ginger, red radish, and garlic?). For each question, the participant received 1 point for the correct answer and 0 points for an incorrect answer; the total score ranged from 0 to 10. Higher scores were indicative of greater gardening knowledge.

#### 2.3.2. Vegetable Consumption

To investigate daily vegetable consumption, a dietary record sheet was developed to note the amount of food consumed based on the 2010 Dietary Reference Intakes proposed by the Korean Nutrition Society. The contents of the record sheet included the type and quantity of foods eaten during the day and how to measure and record food consumption. Before and after the program, children were instructed to record two days’ meals with their parents. Based on the dietary record sheet, the type and amount of food consumed were entered into the CAN 4.0 program developed by the Korean Nutrition Society to calculate the frequency of vegetable consumption. The average vegetable consumption frequency two days before and after the program was calculated and the values analyzed.

#### 2.3.3. Satisfaction Survey

The researchers revised the questionnaires developed by Park et al. [43] and Lee [44] to investigate the participants’ satisfaction with the program. The satisfaction questionnaire consisted of six items: overall satisfaction, satisfaction with program time, satisfaction with program frequency, preferred activities, re-participation, and possibility of recommending the program to others.

We also developed a satisfaction questionnaire for parents and teachers. It consisted of the following items: overall satisfaction, satisfaction with education and newsletter contents, re-participation, possibility of recommending the program to others, whether the children’s eating behavior improved after the program, and so on.

### 2.4. Data Analysis

To compare and analyze the mediating factors related to eating behavior for vegetables before and after the program, a paired *t*-test was conducted. Cronbach’s alpha was also calculated to test the reliability of the evaluation tools. Pearson’s correlation analysis was conducted to examine the correlations between mediating factors for improving eating behavior. A correlation coefficient of less than ± 0.2 means little correlation, that of ± 0.2 to ± 0.4 means low correlation, that of ± 0.4 to ± 0.7 means somewhat high correlation, and that of more than ± 0.7 means high correlation [45]. All statistical analyses were conducted using SPSS version 24 for Windows (IBM, Armonk, NY, USA) and the level of statistical significance was set at *p* < 0.05. Satisfaction with the program and demographic information were analyzed using spreadsheet software in Excel (Office 2016; Microsoft Crop., Redmond, WA, USA).

## 3. Results

### 3.1. Changes in Mediating Factors Related to Eating Behavior for Vegetables

After the program, the self-efficacy, outcome expectancies, gardening knowledge, nutrition knowledge, and vegetable preference scores of all participating children significantly improved (*p* < 0.001; Table 1). Furthermore, the food neophobia scores of all participating children significantly decreased (*p* < 0.05).

Grade-level analysis of gardening knowledge, nutrition knowledge, outcome expectancies for vegetable consumption, self-efficacy, and vegetable preference showed significantly improved scores for both grades (*p* < 0.05; Table 1). However, grade-level analysis of food neophobia showed that the scores of 3rd graders significantly decreased (*p* < 0.01), but there were no significant differences in the scores of 6th graders (*p* > 0.05).

### 3.2. Changes in Vegetable Consumption

After the program, the vegetable consumption frequency of all participating children significantly improved (*p* < 0.001; Table 2). Moreover, grade-level analysis of the vegetable consumption significantly improved scores for both grades (*p* < 0.01; Table 2).

### 3.3. Correlation between Changes in Mediating Factors Related to Eating Behavior for Vegetables

**Figure 3:**
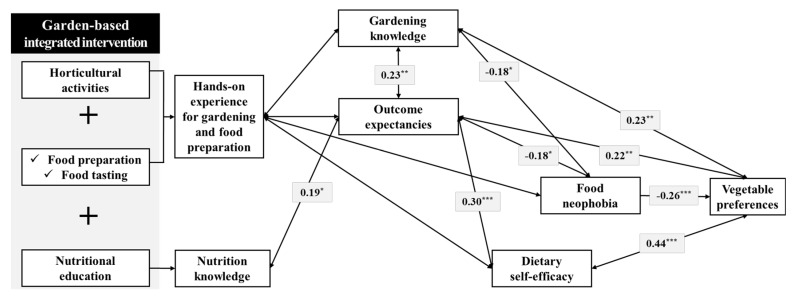
A multiple mediator model derived from analysis of correlation between changes in children’s eating behavior before and after the garden-based integrated intervention. A correlation coefficient of less than ± 0.2 means little correlation, that of ± 0.2 to ± 0.4 means low correlation, that of ± 0.4 to ± 0.7 means somewhat high correlation, and that of more than ± 0.7 means high correlation [45].

The results of correlation analysis between changes in mediating factors related to eating behavior before and after the program presented in Figure 3. The variables that showed a positive correlation with dietary self-efficacy were outcome expectancies (correlation coefficient: 0.30) and vegetable preferences (correlation coefficient: 0.44). The outcome expectancies variable was found to have positive correlations with all other variables (Figure 3). Food neophobia was found to have positive or negative correlations with outcome expectancies, gardening knowledge, and vegetable preferences, which means that these factors have positive effects in reducing children’s food neophobia. Gardening knowledge and vegetable preferences showed a positive correlation with each other (correlation coefficient: 0.23). Accordingly, this result means that all factors in the mediating model developed in this study were found to be statistically positively correlated. In other words, this result means that the mediating factors in this study may have a mutual influence on each other. Ultimately, this interaction of mediating variables may have been affected by the increased vegetable intake of children.

### 3.4. Satisfaction with the Program

After completing the program, the participants and their parents and teachers were asked about their satisfaction with the program. Of the participants, 89.7% (174 students) were satisfied with the overall program and more than half were satisfied with the per session and sessions per week. The most preferred activity was cooking with the harvests (68.0%). Harvesting was the next most popular activity, at 8.8%. The next preferred activities were watering (8.8%), planting (7.7%), making a garden plot (2.1%), managing the garden (2.1%), weeding (2.1%), and sowing seeds (0.5%). Additionally, 81.9% (158 students) answered that they hoped for a continuation of the program and 83.9% (162 students) responded that they would recommend the program to friends.

According to parents’ overall program satisfaction, 75.3% (113 people) answered that they were satisfied. Regarding the program contents, 87.3% (130 people) responded that they were satisfied with the horticultural activities, cooking activities, and nutrition education. Regarding post-program changes, 84.4% (130 people) answered that there were positive changes in their children’s eating behavior.

Regarding teachers, all indicated that they were very satisfied with the overall program. In addition, all responded that they were satisfied with the professionalism and attitude of the instructor, the per session, and sessions per week of the program. Additionally, all answered that there were positive changes in their students’ eating behavior after the program.

## 4. Discussion

This study was to investigate the effects of a garden-based integrated intervention for children’s eating behavior to vegetables and to identify the mediating factors that affect eating behavior for vegetables in children. As the result of the program, dietary self-efficacy, outcome expectancies, gardening knowledge, nutrition knowledge, vegetable preference, and vegetable consumption were significantly increased, and food neophobia was significantly decreased (Table 1 and Table 2). Moreover, there were positive correlations between most mediating factors (Figure 3).

The garden-based integrated intervention significantly increased dietary self-efficacy of children who participated in this study (Table 1). Moreover, as a result of correlation analysis among mediating factors related to improving eating behavior, dietary self-efficacy showed a positive correlation with outcome expectancies and vegetable preferences (Figure 3). This is a similar result to the survey reporting that self-efficacy was positively correlated with positive outcome expectancies in children and adults [46,47,48]. Resnicow et al. [49] reported that children with high dietary self-efficacy for fruit and vegetable selection are more likely to consume fruit and vegetable, with a strongly positive outcome expectancy for fruit and vegetable consumption. Self-efficacy refers to the confidence or expectation of an individual’s ability to organize and directly carry out a series of behavioral processes to achieve a certain outcome [50,51], and it has been identified as an important variable in the study of behavioral changes in children [38,52]. This is developed through direct experience, observation, praise, and persuasion [37], and provides the confidence to overcome temptations that occur in the course of behavioral change or in adopting a new course of behavior [53]. In this study, as children continued to set and achieve vegetable consumption goals, dietary self-efficacy is thought to have improved by enhancing individuals’ beliefs in their capabilities to perform the specific actions required to attain the desired outcome.

Outcome expectancies related to vegetable consumption of children significantly increased after the garden-based integrated intervention (Table 1). The outcome expectancies were negatively correlated with food neophobia and positively correlated with dietary self-efficacy, nutritional knowledge, gardening knowledge, and vegetable preferences (Figure 3). The outcome expectancies variable has been presented as an important determinant of behavioral performance in social cognitive theory and has been reported to be associated with vegetable consumption [49]. Outcome expectancies have been reported to demonstrate positive correlations with dietary self-efficacy, vegetable preferences, fruit and vegetable knowledge, and fruit and vegetable consumption [38,54]. The outcome expectation is the recognition of the consequences of a behavior, and the greater advantages and fewer disadvantages they associate with it, the more people perform that behavior [37,46]. As the visualization of the outcome is based on a positive image related to goal achievement and success, this could serve as motivation for achieving the behavioral objective [55]. In this study, children had hands-on experience in the garden for growing vegetables so that they could increase outcome expectancies for vegetable. Moreover, children learned about the benefits of eating vegetables through nutrition education, thus recognizing the advantages of increasing their vegetable consumption. Furthermore, they learned how to cook the vegetables they had grown, and these factors seemed to raise positive outcome expectancies and decrease negative outcome expectancies.

Children’s food neophobia scores significantly decreased after participating in the garden-based integrated intervention (Table 1). Moreover, food neophobia showed a negative correlation with outcome expectancies, gardening knowledge, and vegetable preferences. Birch et al. [56] and Loewen and Pliner [57] reported that food preferences could be increased simply by increasing food familiarity. Further, Lakkakula et al. [58] and Wardle et al. [59] reported that familiarity with vegetables enables children to accept vegetables positively, resulting in increased vegetable consumption. Food neophobia refers to a refusal to try new foods [40]. As children’s food neophobia results in unhealthy eating habits, such as reduced vegetable preferences and consumption [60,61,62], the reduction of neophobia associated with vegetables is used to facilitate the formation of healthy eating habits in childhood [63]. Providing repeated exposure and positive experiences with new foods and vegetables is an effective strategy to reduce food neophobia in children [3,64]. Especially, practical approaches such as gardening and cooking activities have been shown to increase children’s vegetable consumption more effectively than passive and controlled nutrition education and have a positive impact on their understanding of foods and long-term eating habits [11,38,65]. In this study, sensory stimulation, including seeing, touching, and feeling vegetables directly, improved children’s familiarity with foods, thus potentially reducing neophobia and enhancing the willingness to taste new foods by offering a positive experience [66,67].

Children’s gardening and nutrition knowledge scores improved after the garden-based integrated intervention. Gardening can be an effective strategy to improve children’s knowledge of horticulture and increase their vegetable consumption by involving them in the process of planting, growing, harvesting, and preparing vegetables [68]. Previous studies have shown that repeated positive experiences with vegetables through gardening activity result in children gaining knowledge about vegetables and horticulture, thereby increasing vegetable preferences and consumption [14,15,16,17,69]. Studies have also reported that active participation in and direct experience of gardening programs, including nutrition education, enhance elementary students’ nutrition knowledge and preferences for fruit and vegetable [13,70], and that improved nutrition knowledge has a positive effect on fruit and vegetable consumption [71,72,73]. Schreinemachers et al. [74] reported that school gardening allows children to choose better food and recognize the importance of a healthy diet through their experience of growing vegetables directly at or near schools. Increased nutrition knowledge and the acquisition of vegetable cultivation skills could improve behavioral capability for vegetable consumption from the point of view of social cognitive theory [50,75].

Children’s vegetable preferences and consumption significantly increased (Table 1 and Table 2). This result is similar to Wang’s [76] study, which showed that children who participated in a gardening program including nutrition education and cooking activities had higher fruit and vegetable preferences. In addition, a variety of studies have shown increased vegetable preferences in participants of school garden programs [77,78,79]. This is because through regular exposure to fruit and vegetable by participating in gardening activity, children form positive attitudes toward fruit and vegetable [38,49] and display improved dietary self-efficacy and vegetable preferences [80,81]. Gardening activity provides children with direct exposure to vegetables through the process of growing and harvesting plants and preparing food [26], and this has positive effects including improved preferences for new fruit and vegetable, positive attitude, taste intention, and so on [26,78,82,83]. Therefore, the garden-based integrated intervention affected the factors related to improving children’s eating behavior, which had positive correlations, resulting in improved effects on vegetable consumption.

## 5. Conclusions

In conclusion, the purpose of the present study was to investigate the effects of the garden-based integrated intervention for improving children’s eating behavior for vegetables by measuring the mediating factors related to eating behaviors in children. The result shows the potential of garden-based integrated intervention for improving children’s eating behavior for vegetables. Future study of the topic would be informed by a larger cluster randomized controlled trial to verify the effects of the intervention. Moreover, it would be valuable to verify the lasting effects on eating behaviors through the intervention. Furthermore, it would be interesting to involve parents who have a direct impact on their eating behaviors in the intervention.

## Figures and Tables

**Table 1:** Comparison of mediating factors related to eating behavior in children before and after the garden-based integrated intervention.

Variable		3rd Grade	6th Grade	Total
		Mean ± SD ^1^		
Dietary self-efficacy	*N*	87	96	183
	Pre-test	34.77 ± 8.37	39.24 ± 7.95	37.11 ± 8.43
	Pos*t*-test	40.01 ± 8.33	40.79 ± 7.52	40.42 ± 7.90
	Significance ^2^	(0.000) ***	(0.003) **	(0.000) ***
Outcome expectancies	*N*	84	97	181
	Pre-test	25.60 ± 4.95	27.19 ± 4.68	26.45 ± 4.85
	Pos*t*-test	28.02 ± 5.44	28.01 ± 5.04	28.02 ± 5.21
	Significance	(0.000) ***	(0.042) *	(0.000) ***
Gardening knowledge	*N*	92	98	190
	Pre-test	2.09 ± 1.36	3.50 ± 1.57	2.82 ± 1.63
	Pos*t*-test	4.38 ± 1.90	5.22 ± 1.95	4.82 ± 1.97
	Significance	(0.000) ***	(0.000) ***	(0.000) ***
Nutrition knowledge	*N*	92	98	190
	Pre-test	7.35 ± 2.96	9.91 ± 4.76	8.67 ± 4.18
	Pos*t*-test	9.82 ± 2.95	12.97 ± 4.06	11.44± 3.89
	Significance	(0.000) ***	(0.000) ***	(0.000) ***
Vegetable preferences	*N*	72	96	168
	Pre-test	84.85 ± 26.66	89.20 ± 23.81	87.33 ± 25.09
	Pos*t*-test	101.82 ± 29.84	95.74 ± 24.74	98.35 ± 27.13
	Significance	(0.000) ***	(0.000) ***	(0.000) ***
Food neophobia	*N*	90	98	188
	Pre-test	6.83 ± 4.47	6.01 ± 3.97	6.40 ± 4.22
	Pos*t*-test	5.74 ± 4.66	5.80 ± 4.01	5.77 ± 4.32
	Significance	(0.009) **	NS (0.493)	(0.014) *

^1^ SD: standard deviation; ^2^ * *p* < 0.05, ** *p* < 0.01, *** *p* < 0.001 by paired sample *t*-test.

**Table 2:** Comparison of vegetable consumption in children before and after the garden-based integrated intervention.

Variable		3rd Grade	6th Grade	Total
		Mean ± SD ^1^		
Daily vegetable consumption (frequency)	*N*	64	50	114
	Pre-test	3.43 ± 1.87	3.72 ± 2.11	3.56 ± 1.97
	Pos*t*-test	5.94 ± 2.59	4.92 ± 2.19	5.49 ± 2.47
	Significance ^2^	(0.000) ***	(0.005) **	(0.000) ***

^1^ SD: standard deviation; ^2^ * *p* < 0.05, ** *p* < 0.01, *** *p* < 0.001 by paired sample *t*-test.
